# Maternal High Fat Diet Affects Offspring’s Vitamin K-Dependent Proteins Expression Levels

**DOI:** 10.1371/journal.pone.0138730

**Published:** 2015-09-18

**Authors:** Stuart Lanham, Felino R. Cagampang, Richard O. C. Oreffo

**Affiliations:** 1 Bone and Joint Research Group, Human Development and Health, Institute of Developmental Sciences, Faculty of Medicine, University of Southampton, Southampton, United Kingdom; 2 Maternal, Pregnancy, and Child Research Group, Human Development and Health, Institute of Developmental Sciences, Faculty of Medicine, University of Southampton, Southampton, United Kingdom; Consiglio Nazionale delle Ricerche, ITALY

## Abstract

Studies suggest bone growth & development and susceptibility to vascular disease in later life are influenced by maternal nutrition, during intrauterine and early postnatal life. There is evidence for a role of vitamin K-dependent proteins (VKDPs) including Osteocalcin, Matrix-gla protein, Periostin, and Gas6, in bone and vascular development. This study extends the analysis of VKDPs previously conducted in 6 week old offspring, into offspring of 30 weeks of age, to assess the longer term effects of a maternal and postnatal high fat (HF) diet on VKDP expression. Overall a HF maternal diet and offspring diet exacerbated the bone changes observed. Sex specific and tissue specific differences were observed in VKDP expression for both aorta and femoral tissues. In addition, significant correlations were observed between femoral *OCN*, *Periostin Gas6*, and *Vkor* expression levels and measures of femoral bone structure. Furthermore, *MGP*, *OCN*, *Ggcx* and *Vkor* expression levels correlated to mass and fat volume, in both sexes. In summary the current study has highlighted the importance of the long-term effects of maternal nutrition on offspring bone development and the correlation of VKDPs to bone structure.

## Introduction

The vitamin K dependent proteins (VKDPs) consist of at least 16 known proteins with varied roles, including factors involved in blood coagulation (such as factors VII and X) and anti-coagulation (e.g. proteins C and S). However, other VKDPs have emergent roles in other organs whilst four VKDPs have been reported to play key roles in both bone and vascular tissues, with alterations in expression resulting in disease pathologies [1].

There is now evidence that bone can act as an endocrine organ, influencing other organs, with Osteocalcin (OCN) implicated as a key player in this process [2–4]. OCN is a hormone secreted by osteoblasts that increases insulin sensitivity and production thus enhancing glucose utilisation and energy expenditure [5].

Matrix gla protein (MGP) is evolutionarily related to OCN [6], thus may possess hormonal properties. However, MGP is found throughout the body and prevents calcification of tissues such as lung, kidney, coronary arteries and aorta [7]. The VKDP Periostin is important in bone growth [8] and essential for cardiac development [9]. Periostin is present in connective tissues subject to mechanical forces (e.g. heart valves) and is present in glomeruli in patients with nephropathy [10]. Periostin is also highly up-regulated following cardiac tissue injury [8].

Finally, Growth-arrest specific protein 6 (Gas6) regulates a mix of processes, including cell survival and proliferation, cell adhesion and migration, blood clot stabilisation, and inflammatory cytokine release. Recently, *Gas6* expression was shown to regulate chondrogenic differentiation [11] and to be associated with altered glucose tolerance, inflammation [12], small for gestational age [13] as well as cardiovascular disease [14]. In addition to these VKDPs, vitamin K itself has been shown to be key in coagulation as well as to play a number of roles in bone growth and development [15]. A recent study in dialysis patients suggests vitamin K deficiency as a possible cause of both vertebral fractures and vascular calcification [16].

In addition, at least two enzymes are required for VKDP function; i) gamma-glutamyl carboxylase (Ggcx), which mediates the vitamin K-dependent carboxylation of glutamate residues to calcium-binding gamma-carboxyglutamate (Gla) residues, thereby converting vitamin K hydroquinone to epoxide and, ii) Vitamin K epoxide reductase (Vkor), which reduces inactive vitamin K epoxide to active vitamin K hydroquinone via vitamin K [17].

Epidemiological and animal studies indicate that environmental factors, such as maternal nutrition, influence disease risk in later life [18–21]. Indeed, numerous studies have shown that intrauterine growth restriction, a proxy measure of poor prenatal environment, can affect cardiovascular and metabolic control in animals and humans in a nutritionally abundant postnatal environment [22–25]. Furthermore, dysfunction of the vascular system can lead to a number of diseases including hypertension, atherogenesis, type 2 diabetes, coronary heart disease, metabolic syndrome, and obesity. Critically, the risk and development of these diseases has been shown to be influenced by maternal nutrition during pregnancy; commonly referred to as the developmental origins concept [20]. The underlying mechanisms are thought to involve epigenetic modifications, resulting in life-long alterations in gene expression [26].

Previously we have assessed the effect of maternal and offspring high fat diet on VKD proteins in the aorta and bone from 6 week old mice [27]. The data presented in this study extends these studies with analysis performed in offspring at 30 weeks of age.

## Materials and Methods

### Experimental Design and Animal Care

All mice were raised within the University of Southampton Biomedical Research Facility and were housed in appropriate environments in rooms maintained at 22 ± 2°C with a 12 h light:12 h dark cycle.

### Ethical Statement

All animal procedures were in accordance with the regulations of the United Kingdom Animals (Scientific Procedures) Act 1986 and were conducted under Home Office Project Licence number 30–2968. The study received institutional approval from the University of Southampton Biomedical Research Facility Research Ethics Committee.

### High Fat Diet

At 10 weeks of age, 11 female C57BL/6 strain mice were mated with aged-matched C57BL/6 males. Females were mated with separate males, and after confirmation of mating (presence of a vaginal plug) were individually housed and fed either a standard chow control (C, n = 5 dams) diet RM-1 (Special Diet Services, Witham, Essex, UK) or a high-fat (HF, n = 6 dams) diet (Special Diet Services diet 824053, Special Diet Services). We have used these diets in an earlier study [27]. These C or HF diets were continued throughout pregnancy and lactation until the offspring had been weaned at 3 weeks of age. The weaned offspring were then further subdivided to provide each parental dietary group with offspring that had been fed either the control (C/C or HF/C groups) or the high-fat diet (C/HF or HF/HF groups). Same sex littermates on the same post-weaning diet were housed together. For all groups n = 10–12 animals, with n = 5–7 males or females per group. This diet was continued for another 27 weeks up to the time of sampling at 30 weeks of age. Animals were killed by cervical dislocation.

### 3D Computed Tomography

Whole animals were scanned using a Skyscan 1176 *in vivo* micro-CT scanner (Bruker microCT, Kontich, Belgium). All scans were taken at 50 kV, 500 μA with 0.5 mm aluminium filter, with 0.5° rotation step. Individual 2D cross-sectional images were reconstructed using Bruker NRecon software version 1.6.5.8. Voxel resolution was 18 μm. Reconstructed images were analysed using Bruker CTAn software version 1.13.5.1 with appropriate thresholds to determine volumes of fat, soft tissue, and bone. For the vertebra, the vertebral body of L3 was analysed. For the femur, a section 0.35mm in height and 1.8mm behind the growth plate at the distal end was analysed.

The trabecular parameters measured were BvTv (volume of bone within a measured total volume), BsBv (surface to volume ratio of trabecular bone), trabecular thickness, trabecular spacing (distance between trabeculae), structural model index (SMI, measure of surface convexity where a lower SMI is indicative of more connected, plate-like trabeculae), trabecular pattern factor (indicator of connectivity of trabeculae where values closer to zero, both positive or negative, represent a more connected structure).

### Sample Collection

#### Mice

Following the CT scan (see above), the thoracic aorta and left femur were removed. The left femur was cleaned of soft tissues, cut in half, and the marrow was flushed out using PBS through a fine gauge needle. The halves were then cut into smaller pieces. Following processing, the aorta and femur bone pieces were immediately placed in Trizol Reagent (Invitrogen).

### RNA Extraction and cDNA Production

RNA was extracted from samples in Trizol Reagent (Invitrogen) according to manufacturer’s instructions. The concentration and purity of RNA was determined by optical densities at 230 nm, 260 nm and 280 nm using a NanoDrop Spectrophotometer (Labtech, Uckfield, UK), and cDNA produced from 500ng RNA using Superscript VILO cDNA synthesis kit (Invitrogen) following manufacturer’s instructions.

### Quantitative PCR

Relative quantification of gene expression was performed with an ABI Prism 7500 detection system (Applied Biosystems, Warrington, UK). The 20 μl reaction mixture was prepared, containing 1 μl of complementary DNA, 10 μl of Power SYBR Green PCR Master Mix (Applied Biosystems), and 250 nM of each primer. Thermal cycler conditions consisted of an initial activation step at 95°C for 10 min, followed by a 2-step PCR program of 95°C for 15 s and 60°C for 60 s for 40 cycles. A dissociation curve was obtained for each run. The 2^−ΔΔCt^ method was employed for relative quantification of gene expression compared to the male C/C group, and the data were normalized to glyceraldehyde-3-phosphate dehydrogenase (*Gapdh*) expression (which was unchanged between groups). The mouse primers used for qPCR were: *Gapdh* forward, AGGTCGGTGTGAACGGATTTG; reverse, TGTAGACCATGTAGTTGAGGTCA; *Mgp* forward, TCAACAGGAGAAATGCCAACAC; reverse, CGGTTGTAGGCAGCGTTGT; *OCN* forward, CTGACCTCACAGATGCCAAGC; reverse, TGGTCTGATAGCTCGTCACAAG; *Gas6* forward, TGCTGGCTTCCGAGTCTTC; reverse, CGGGGTCGTTCTCGAACAC; *Periostin* forward, CCTGCCCTTATATGCTCTGCT; reverse, AAACATGGTCAATAGGCATCACT; *Ggcx* forward, GTTGCTCCCGCCTCAGATAAA; reverse, TAAGCAGGGTCACGACACTCT; *Vkor* forward, GCTGGCTTAGCCCTCTCAC; reverse, CTGTCCGCTCCTAGCATGT.

### Statistics

All data for all diet groups was observed to be normally distributed using the Shapiro-Wilks test. The effect of maternal, offspring diet and sex was determined by three-way ANOVA followed by Bonferroni post hoc test using PASW version 21 (SPSS UK, Woking, Surrey, United Kingdom). Data are presented as mean ± Standard Deviation unless otherwise shown; significance was determined with a p-level of 0.05 or lower. At least 4 dams were used for each diet group. Relationships between variables were tested using linear regression analysis (univariant and multivariant) where a p<0.05 was considered statistically significant.

## Results

### Body Mass and Composition

#### Male Offspring

**Fig 1:**
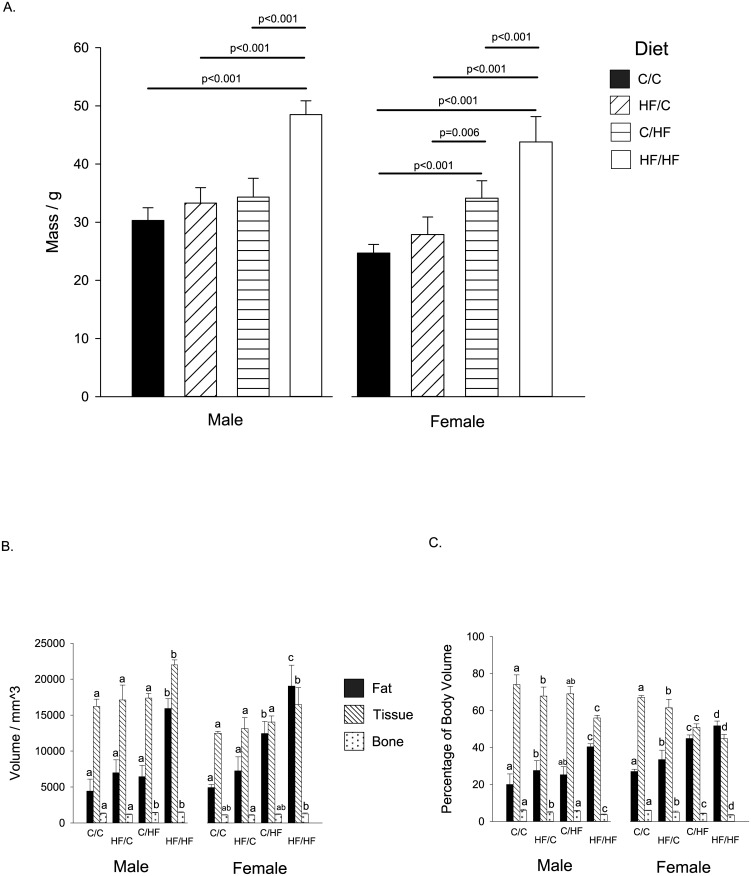
Offspring Mass and body Composition at Thirty Weeks of Age. Graphs show (A). Mass. (B). Fat, Tissue and Bone Volume. (C). Percentage of Body Fat, Tissue and Bone. Within each sex and for each tissue type, bars with different letters are significantly different (p<0.05). For all groups, n = 5–7 per group. Graphs show mean plus 95% confidence limits.

For male offspring, a combination of maternal and offspring high fat diet (i.e. the HF/HF group) significantly increased offspring mass above control levels (C/C group; p<0.001) (Fig 1A left panel). Furthermore, this diet combination significantly increased total fat and tissue volumes above all other offspring groups (All p<0.001) (Fig 1B left panel). In addition, the total bone volume was increased above C/C levels in offspring groups on postnatal HF diet (C/HF p = 0.05, and HF/HF p = 0.01) (Fig 1B left panel). However, the percentage of body fat was increased above C/C levels in offspring groups from dams on HF diet during pregnancy (HF/C p = 0.02, and HF/HF p<0.001) (Fig 1C left panel), with a corresponding decreased in percentage of tissue (HF/C p = 0.03 and HF/HF p<0.001) and bone (HF/C p = 0.001 and HF/HF p<0.001) in these groups (Fig 1C left panel).

The results of univariant linear regression analysis for male offspring are shown in Table 1A. Body mass and volume parameters were observed to be significantly correlated with expression levels of *MGP*, O*CN*, *Periostin*, *Ggcx*, and *Vkor*. Multivariant analysis of these parameters showed a significant correlation only with *OCN*, *Periostin*, *Ggcx* and *Vkor*. Of these, *Periostin*, *Ggcx* and *Vkor* also showed significant correlation with the addition of maternal diet. However, using offspring diet for multivariant analysis showed significant correlation with *OCN*, *Periostin*, *Gas6*, *Ggcx* and *Vkor* expression (Table 1A).

**Table 1:** Linear regression analysis between femoral vitamin K-dependent protein expression levels and body composition.

A. Males																			
Site	Factor	*Mgp*			*Osteocalcin*			*Periostin*			*Gas6*			*Ggcx*			*Vkor*		
		β	r^2^	p	β	r^2^	p	β	r^2^	p	β	r^2^	p	β	r^2^	p	β	r^2^	p
Body	Mass	0.63	0.39	0.007	0.74	0.54	0.001	0.76	0.58	<0.001	-0.31	0.09	ns	0.78	0.61	<0.001	0.84	0.70	<0.001
	Fat volume	0.66	0.43	0.004	0.71	0.51	0.001	0.77	0.59	<0.001	-0.33	0.11	ns	0.78	0.60	<0.001	0.86	0.74	<0.001
	Tissue volume	0.60	0.36	0.01	0.67	0.44	0.004	0.75	0.56	0.001	-0.30	0.09	ns	0.80	0.63	<0.001	0.84	0.70	<0.001
	Bone volume	0.29	0.08	ns	0.28	0.08	ns	0.55	0.30	0.02	-0.12	0.01	ns	0.53	0.28	0.02	0.59	0.35	0.006
	Multivariant	F(4,12) = 2.45		ns	F(4,12) = 3.88		0.03	F(4,12) = 4.59		0.02	F(4,15) = 0.47		ns	F(4,14) = 6.32		0.004	F(4,15) = 14.72		<0.001
	+ Maternal diet	F(5,11) = 1.93		ns	F(5,11) = 3.00		0.06	F(5,11) = 3.96		0.03	F(5,14) = 0.65		ns	F(5,13) = 5.66		0.005	F(5,14) = 11.48		<0.001
	+ Offspring diet	F(5,11) = 1.86		ns	F(5,11) = 12.82		<0.001	F(5,11) = 4.73		0.02	F(5,14) = 3.57		0.03	F(5,13) = 4.74		0.01	F(5,14) = 11.03		<0.001
B. Females																			
Site	Factor	*Mgp*			*Osteocalcin*			*Periostin*			*Gas6*			*Ggcx*			*Vkor*		
		β	r^2^	p	β	r^2^	p	β	r^2^	p	β	r^2^	p	β	r^2^	p	β	r^2^	p
Body	Mass	0.70	0.49	0.001	0.58	0.34	0.006	-0.16	0.03	ns	0.26	0.07	ns	0.74	0.55	<0.001	0.78	0.61	<0.001
	Fat volume	0.70	0.49	0.001	0.60	0.35	0.004	-0.22	0.05	ns	0.25	0.06	ns	0.73	0.53	<0.001	0.72	0.52	<0.001
	Tissue volume	0.52	0.27	0.03	0.41	0.17	0.08	-0.06	0.00	ns	0.17	0.03	ns	0.59	0.35	0.005	0.73	0.53	<0.001
	Bone volume	0.69	0.48	0.001	0.18	0.03	ns	0.01	0.00	ns	0.31	0.05	ns	0.54	0.29	0.01	0.52	0.27	0.02
	Multivariant	F(4,13) = 11.37		<0.001	F(5,15) = 3.48		0.03	F(4,12) = 0.99		ns	F(4,14) = 0.80		ns	F(4,16) = 5.99		0.004	F(4,14) = 7.88		0.002
	+ Maternal diet	F(5,12) = 8.42		0.001	F(5,14) = 2.92		0.05	F(5,11) = 3.22		0.05	F(5,13) = 0.60		ns	F(5,15) = 7.39		0.001	F(5,13) = 9.82		<0.001
	+ Offspring diet	F(5,12) = 8.40		0.001	F(5,14) = 3.47		0.03	F(5,11) = 2.11		ns	F(5,13) = 0.66		ns	F(5,15) = 7.72		0.001	F(5,13) = 5.95		0.004

For Diet, β value is based on high fat diet having a higher value than control diet.

#### Female Offspring

For female offspring, an offspring HF diet, either alone or in combination with a maternal HF diet, increased offspring mass above control levels (C/HF or HF/HF, both p<0.001) (Fig 1A right panel). In addition, offspring mass was significantly increased with a maternal HF diet compared to a control maternal diet for females fed an offspring HF diet (C/HF v HF/HF, p<0.001) (Fig 1A right panel). The offspring groups on postnatal HF diet also resulted in significant increases in total fat volumes versus controls (C/HF or HF/HF v C/C, both p<0.001) (Fig 1B right panel), with significantly higher volume in the HF/HF group compared to the C/HF group (p<0.001). Total tissue volume was only significantly increased above C/C levels in the HF/HF group (p<0.001) (Fig 1B right panel). Critically, total bone volume was not significantly increased above C/C levels in all other offspring groups, however, the HF/HF group showed an increase in bone volume compared to the HF/C group (p = 0.01) (Fig 1B right panel). The percentage of fat was significantly increased versus C/C in all other offspring groups (all p<0.003) (Fig 1C right panel), with greater increases in the offspring fed postnatally the HF diet (C/HF and HF/HF), and a further significant increase in the HF/HF group compared to C/HF group (p = 0.001). As a consequence, this produced corresponding significant decreases in both total tissue and bone volumes in all the offspring groups versus C/C females (all p<0.008) (Fig 1C right panel).

The results of univariant linear regression analysis for female offspring are shown in Table 1B. Body mass and volume parameters were significantly correlated with expression levels of *MGP*, O*CN*, *Ggcx* and *Vkor*. Multivariant analysis of these parameters showed a significant correlation with *MGP*, *OCN*, *Ggcx* and *Vkor* (with or without the addition of maternal and offspring diets). Periostin only showed significant correlations with multivariant analysis with maternal diet (Table 1B).

### Analysis of the Bone Structure of the Femur

#### Male Offspring

**Fig 2:**
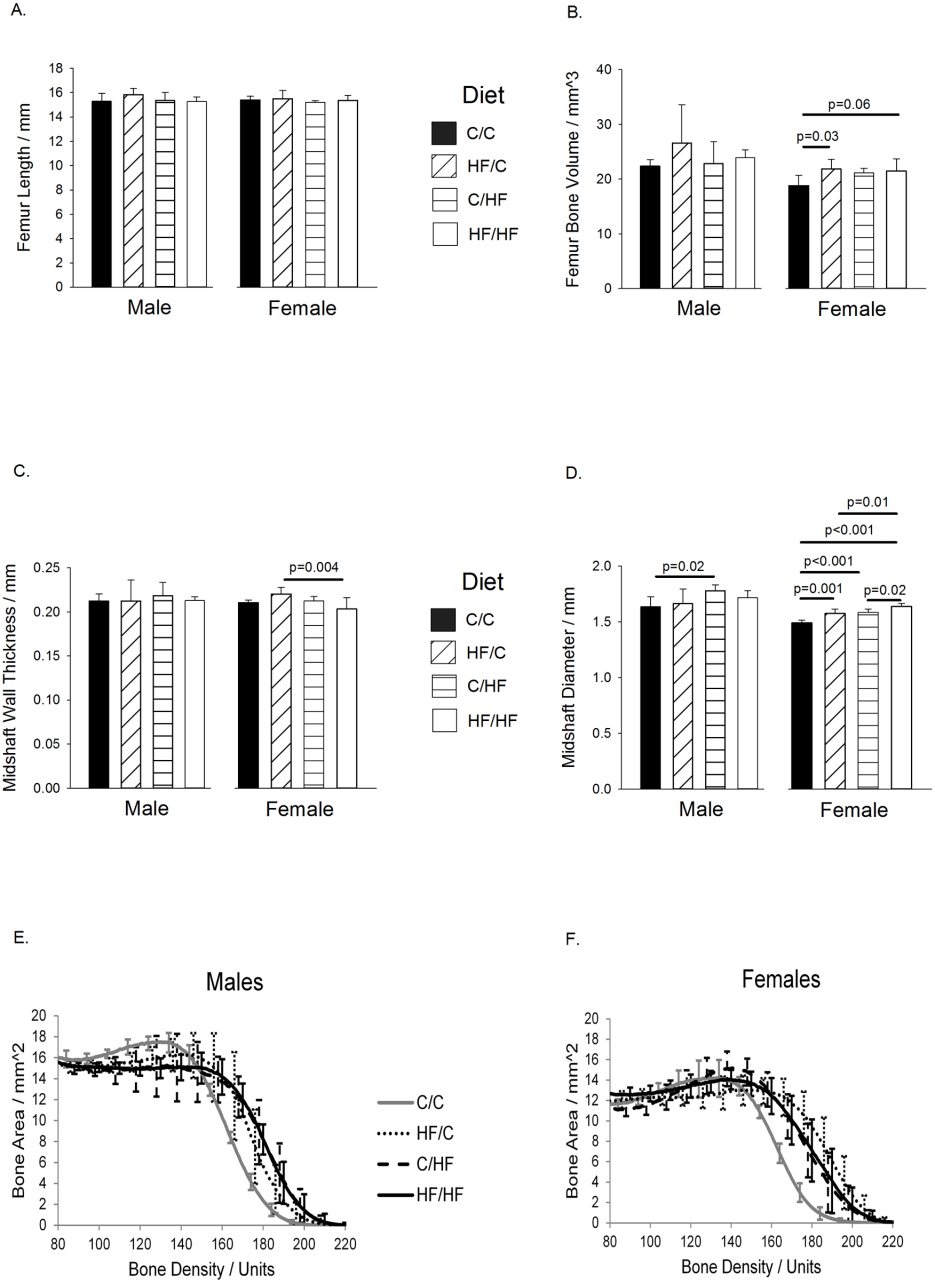
Femoral Bone Structure at Thirty Weeks of Age. For dietary group and sex, results are shown for (A). Femur length. (B). Femur bone volume. (C). Midshaft wall thickness. (D). Midshaft diameter. (E). Male femur bone density. (F). Female femur bone density. For density graphs, the bone density range 80–140 represents trabecular bone, and 140–220 represents cortical bone. For all groups, n = 5–7 per group. Graphs show mean plus 95% confidence limits.

The left femur was analysed in all samples (Fig 2). For male offspring, no significant increase in femur length (Fig 2A left panel) or femur bone volume (Fig 2B left panel) were found between any of the diet groups. Furthermore, there was no difference in femoral midshaft wall thickness between any of the male offspring groups (Fig 2C left panel). The male C/HF group showed a significant increase in femoral midshaft diameter compared to controls (Fig 2D left panel). Compared to controls, the HF/HF group showed a significant reduction in femoral trabecular bone volume to total volume ratio (Table 2) and a corresponding increase in trabecular spacing (Table 2). The HF/HF also showed increased structural model index (Table 2) indicated a more rod-like trabecular structure than controls. The increased trabecular pattern factor seen in the HF/HF group (Table 2) indicated reduced trabecular connectivity compared to controls. The HF/C groups showed increased trabecular spacing compared to controls (Table 2). For the femoral bone density, the C/HF and HF/HF groups showed a significantly higher bone area, compared to controls, for the range 170–180 (p<0.05, Fig 2E), and the HF/HF group were observed to be higher than controls at densities of 160 and 190 although this was not significant (p<0.07, Fig 2E). These observations indicate an increase in cortical bone in these groups. No consistent differences were seen in the trabecular bone region density range of 80–140 (Fig 2E).

**Table 2:** Bone structural parameters for femur and 3^rd^ lumbar vertebra from 30 week old mice.

	Males				Females			
	C/C	HF/C	C/HF	HF/HF	C/C	HF/C	C/HF	HF/HF
	(n = 5)	(n = 6)	(n = 5)	(n = 7)	(n = 5)	(n = 6)	(n = 6)	(n = 7)
Femur	Mean (SD)	Mean (SD)	Mean (SD)	Mean (SD)	Mean (SD)	Mean (SD)	Mean (SD)	Mean (SD)
BvTv	27.1 (2.3)^a^	22.3 (10.0)^ab^	20.5 (4.2)^ab^	13.5 (4.2)^b^	4.2 (1.4)	6.0 (1.2)	5.9 (1.8)	5.1 (0.7)
BsBv	36.9 (2.1)	38.4 (11.7)	37.8 (6.3)	38.8 (5.0)	55.3 (4.0)^a^	49.2 (2.8)^ab^	45.7 (4.9)^b^	47.3 (3.5)^b^
TbTh / mm	0.099 (0.006)	0.106 (0.021)	0.102 (0.013)	0.103 (0.012)	0.079 (0.004)	0.085 (0.005)	0.091 (0.009)	0.091 (0.008)
TbSp / mm	0.200 (0.017)^a^	0.282 (0.033)^b^	0.264 (0.040)^ab^	0.381 (0.049)^c^	0.437 (0.039)	0.425 (0.044)	0.477 (0.043)	0.461 (0.069)
SMI	1.6 (0.1)^a^	2.1 (0.5)^ab^	2.0 (0.3)^ab^	2.4 (0.2)^b^	2.9 (0.2)	2.7 (0.1)	2.8 (0.3)	2.8 (0.1)
TbPF / mm^-1^	4.9 (1.3)^a^	12.2 (8.2)^ab^	9.9 (3.7)^ab^	13.9 (3.8)^b^	26.7 (3.9)^a^	21.7 (1.8)^b^	20.2 (2.1)^b^	23.0 (2.4)^ab^
Vertebra								
BvTv	43.7 (3.3)^a^	28.9 (1.9)^b^	40.1 (11.5)^ab^	36.0 (3.8)^ab^	25.6 (3.0)^a^	31.1 (6.7)^ab^	35.3 (1.4)^b^	31.5 (3.1)^ab^
BsBv	35.6 (2.1)^a^	42.7 (2.7)^a^	35.2 (5.8)^a^	36.5 (3.2)^a^	41.2 (2.3)^a^	37.0 (2.3)^b^	31.3 (0.6)^c^	34.4 (1.2)^b^
TbTh / mm	0.093 (0.004)^a^	0.083 (0.006)^a^	0.097 (0.011)^a^	0.092 (0.008)^a^	0.086 (0.003)^a^	0.092 (0.005)^a^	0.108 (0.003)^b^	0.099 (0.003)^c^
TbSp / mm	0.148 (0.009)^a^	0.183 (0.009)^b^	0.168 (0.030)^ab^	0.177 (0.007)^b^	0.300 (0.023)^a^	0.246 (0.051)^a^	0.250 (0.012)^a^	0.251 (0.027)^a^
SMI	0.8 (0.2)^a^	1.6 (0.1)^b^	1.1 (0.7)^ab^	1.2 (0.2)^ab^	1.6 (0.2)^a^	1.2 (0.4)^a^	1.2 (0.2)^a^	1.3 (0.1)^a^
TbPF / mm^-1^	-0.7 (1.9)^a^	8.0 (0.9)^b^	1.8 (6.0)^ab^	3.4 (2.0)^ab^	7.3 (1.8)^a^	3.9 (0.4)^ab^	3.3 (1.1)^b^	4.4 (1.0)^ab^

Abbreviations are BvTv: trabecular bone volume to total volume, BsBv: Bone surface to bone volume ratio, TbTh: Trabecular thickness, TbSp: Trabecular spacing, SMI: Structural model index, TbPF: Trabecular pattern factor. ^a, b,c^: For each parameter measured, values with different superscript letters are significantly different from each other (p<0.05) within the same sex.

#### Female Offspring

No differences were seen in femur length between any of the female offspring groups (Fig 2A right panel). In contrast, both maternal HF diet groups (HF/C, p = 0.03 and HF/HF, p = 0.06) showed increases in femur bone volume compared to C/C control females (Fig 2B right panel). The femoral midshaft wall thickness was thicker in the female HF/C group although this was only significant compared to the HF/HF group (Fig 2C right panel). The femoral midshaft diameter was significantly increased in all female diet groups compared to controls (Fig 2D right panel), with the female HF/HF group displaying a significantly larger diameter than the HF/C and C/HF groups. The offspring HF diet groups (C/HF and HF/HF) showed reduced femoral bone surface to volume ratio than controls (Table 2), however, the corresponding increase in trabecular thickness was not statistically significant (Table 2). For the femoral bone density, the HF/C group showed a significantly higher bone area, compared to controls, for the range 160–190 (p<0.05, Fig 2F), the C/HF group showed a significantly higher bone area for the range 150–170 (p<0.05, Fig 2F), and HF/HF group showed a significantly higher bone area for the range 160–170 (p<0.05, Fig 2F) and HF/HF group showed raised values over controls at 180 density although this was not significant (p<0.06), indicating an increase in cortical bone in these groups (Fig 2F). No differences were seen in the trabecular bone region density range of 80–140 (Fig 2F).

### Analysis of the Bone Structure of the Vertebra

#### Male Offspring

**Fig 3:**
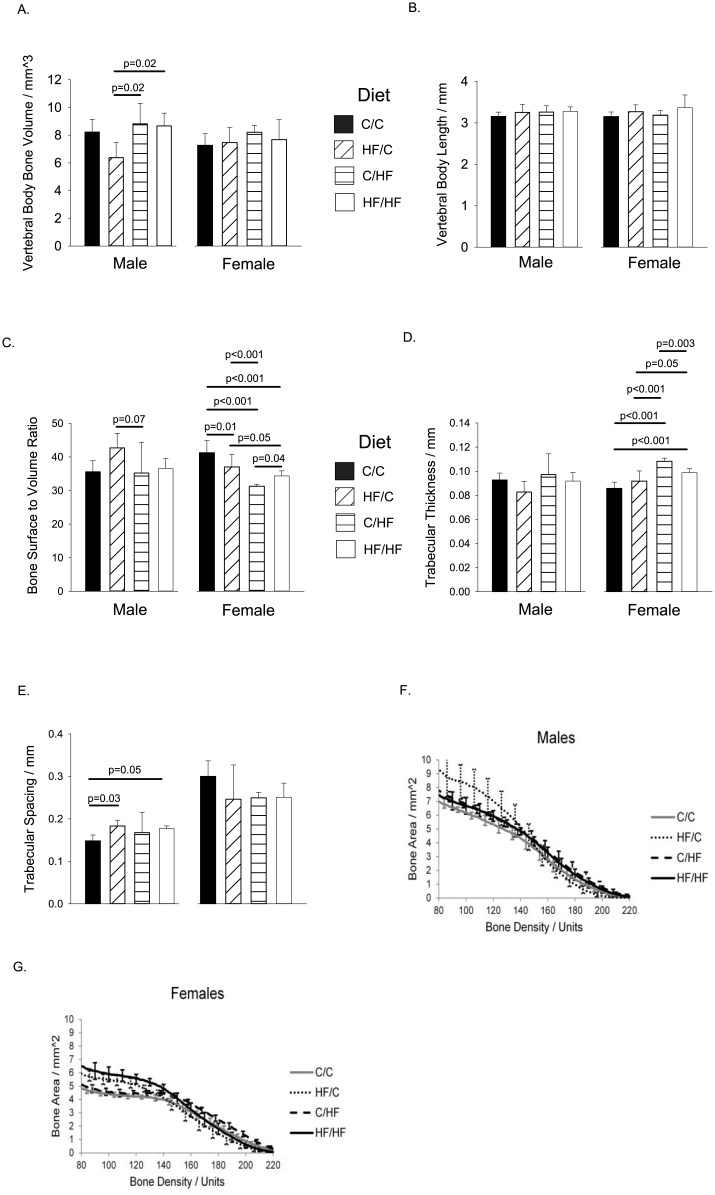
Vertebral Body Bone Structure at Thirty Weeks of Age. For dietary group and sex, results are shown for (A). Vertebral body bone volume. (B). Vertebral body length. (C). Bone surface to volume ratio. (D). Trabecular thickness. (E). Trabecular spacing. (F). Male whole vertebra bone density. (G). Female whole vertebra bone density. For density graphs, the bone density range 80–140 represents trabecular bone, and 140–220 represents cortical bone. For all groups, n = 5–7 per group. Graphs show mean plus 95% confidence limits.

The 3^rd^ lumbar vertebra was analysed in all samples (Fig 3). For male offspring, no significant differences were seen in any of the diet groups in vertebral body length (Fig 3B left panel), trabecular bone surface to volume ratio (Fig 3C left panel), or trabecular thickness (Fig 3D left panel). In contrast, the HF/C group showed reduced vertebral bone volume compared to the offspring high fat diet groups (C/HF and HF/HF) (Fig 3A left panel) the maternal HF diet groups (HF/C and HF/HF) showed significant increases in trabecular spacing compared to C/C controls (Fig 3E left panel). In comparison to C/C controls, the HF/C group showed reduced trabecular bone volume to total volume ratio (Table 2), increased structural model index, and trabecular pattern factor. No consistent differences were seen in bone density between any of the diet groups (Fig 3F).

#### Female Offspring

For female offspring, no significant differences were seen versus C/C controls in any of the other offspring groups in the vertebral bone volume (Fig 3A right panel), vertebral body length (Fig 3B right panel), or trabecular spacing (Fig 3E right panel). However, there was a reduction in trabecular bone surface to volume ratio in all offspring groups versus C/C controls (Fig 3C right panel), the greatest reduction occurring in the C/HF group. There was a corresponding significant increase in trabecular thickness in both offspring groups on postnatal HF diet versus C/C controls (Fig 3D right panel), with the greatest thickness occurring in the C/HF group. Compared to C/C controls, the C/HF offspring also showed increased trabecular bone volume to total volume ratio (Table 2), and reduced trabecular pattern factor. For the bone density, offspring groups from HF-fed dams (HF/C and HF/HF) showed a significantly higher bone area, compared to offspring groups from C-fed dams (C/C and C/HF), for the range 80–120 (p<0.05, Fig 3G), indicating an increase in trabecular bone. No consistent differences were seen in the cortical bone region density range of 140–220 (Fig 3G).

### VKDPs in Aorta

#### Male Offspring

**Fig 4:**
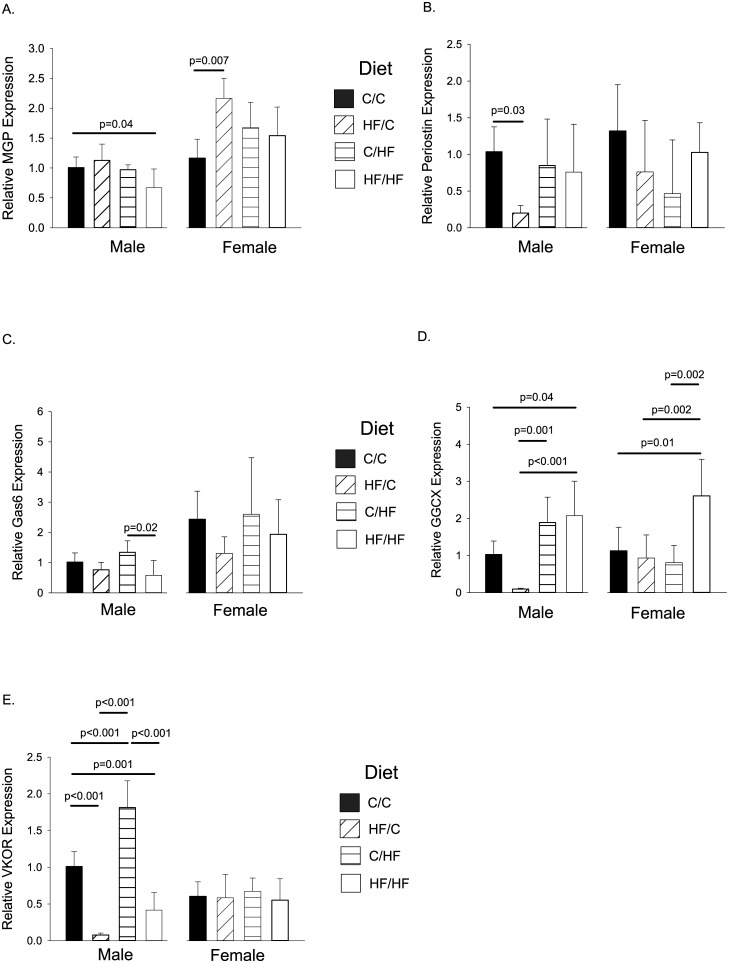
Mean Relative Gene Expression Levels in Aorta for Thirty Week Old Mice. Gene expression was determined by qPCR. Graphs show gene expression for (A). *MGP*, (B). *Periostin*, (C). *Gas6*, (D). *Ggcx*, and (E). *Vkor*. Gene expression is shown relative to male C/C group. For all groups, n = 5–7 per group. Graphs show mean plus 95% confidence limits.

The expression levels of *Mgp*, *Periostin*, *Gas6*, *Ggcx*, and *Vkor* in the aorta are presented in Fig 4 (left panels). *MGP* expression was found to be significantly lower in HF/HF males compared to C/C controls (p = 0.04, Fig 4A left panel). HF/C male offspring were found to have significantly lower expression levels of *Periostin* compared to controls (p = 0.03, Fig 4B left panel). Expression levels of *Gas6* tended to be lower in the offspring groups from HF-fed dams (HF/C and HF/HF, Fig 4C left panel), but this was only significantly different between the C/HF and HF/HF groups (p = 0.02). Compared to C/C controls, *Ggcx* levels were raised in the HF/HF group (p = 0.04, Fig 4D left panel). *Ggcx* expression was significantly reduced in the HF/C offspring compared to both HF offspring groups (p = 0.001 for C/HF, and p<0.001 for HF/HF, Fig 4D left panel). *Vkor* expression was altered in all test groups compared to controls (Fig 4E left panel). Expression levels were significantly lower in both maternal HF-fed groups (p<0.001 for HF/C and p = 0.001 for HF/HF). In contrast, *Vkor* expression levels were significantly raised in the C/HF group (p<0.001, Fig 4E left panel).

#### Female Offspring

The expression levels of *Mgp*, *Periostin*, *Gas6*, *Ggcx*, and *Vkor* in the aorta are presented in Fig 4 (right panels). *MGP* expression levels were significantly higher in HF/C female offspring compared to C/C controls (p = 0.007, Fig 4A right panel). No significant differences were seen in expression levels of *Periostin*, *Gas6*, or *Vkor*. *Ggcx* expression levels were significantly higher in HF/HF offspring compared to all other groups (p = 0.01 for controls, p = 0.002 for HF/C and C/HF, Fig 4D right panel).

### VKDPs in Femoral Bone

#### Male Offspring

**Fig 5:**
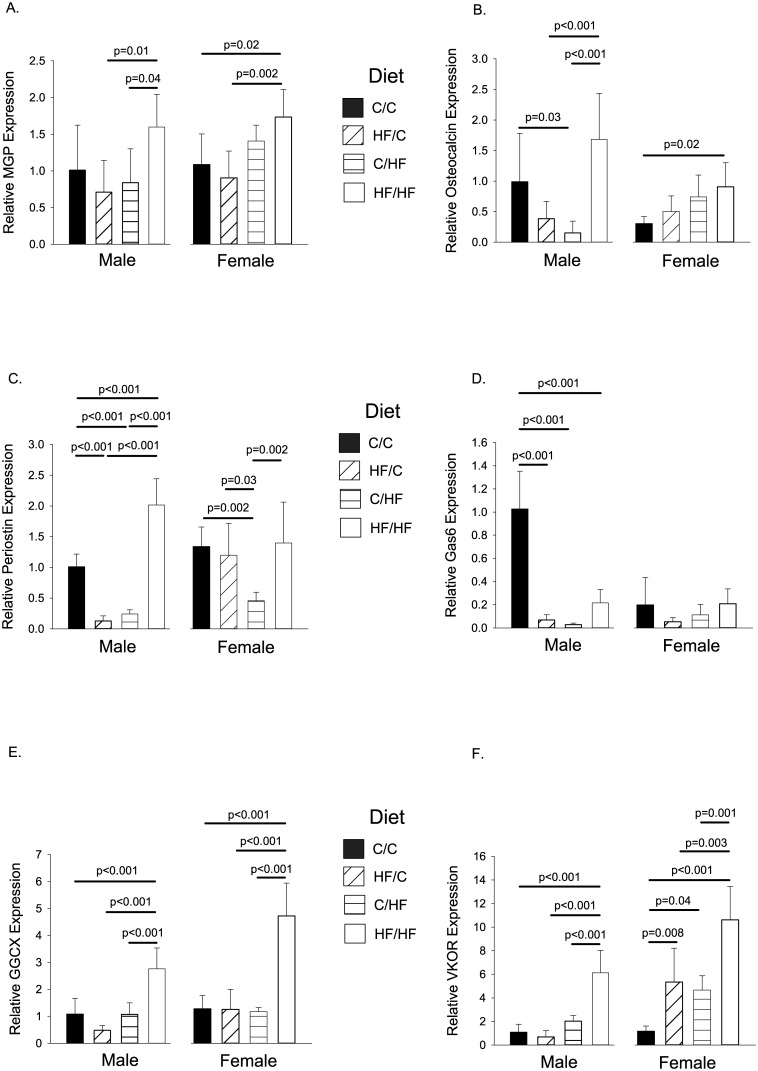
Mean Relative Gene Expression Levels in Femur for Thirty Week Old Mice. Gene expression was determined by qPCR. Graphs show gene expression for (A). *MGP*, (B). *Osteocalcin*, (C). *Periostin*, (D). *Gas6*, (E). *Ggcx*, and (F). *Vkor*. Gene expression is shown relative to male C/C group. For all groups, n = 5–7 per group. Graphs show mean plus 95% confidence limits.

The mRNA expression levels of *Mgp*, *OCN*, *Periostin*, *Gas6*, *Ggcx*, and *Vkor* in the femur are shown in Fig 5 (left panels). MGP expression levels were significantly higher in the HF/HF group compared to HF/C (p = 0.01) and C/HF (p = 0.04) offspring, although not to controls (Fig 5A left panel). *OCN* levels were significantly higher in HF/HF male offspring compared to HF/C and C/HF groups (Both p<0.001, Fig 5B left panel), with C/HF levels also significantly lower than controls (p = 0.03, Fig 5B left panel). *Periostin* levels were significantly reduced in HF/C and C/HF groups (both p<0.001) and higher in HF/HF group (p<0.001) compared to controls (Fig 5C left panel). *Gas6* expression levels were lower in all 3 test diet groups compared to controls (all p<0.001, Fig 5D left panel). The HF/HF male offspring had significantly higher *Ggcx* expression levels than all other diet groups (all p<0.001, Fig 5E left panel). *Vkor* expression levels were significantly higher than controls and the HF/C & C/HF diets in the HF/HF male offspring (all p<0.001, Fig 5F left panel).

#### Female Offspring

*MGP* expression levels were significantly higher in HF/HF female offspring compared to C/C controls (p = 0.02) and HF/C (p = 0.002) groups (Fig 5A right panel). *OCN* levels in female offspring were significantly higher than controls in the HF/HF diet group (p = 0.02, Fig 5B right panel). *Periostin* expression levels were significantly lower in C/HF offspring compared to all other diet groups (p = 0.002 for controls, p = 0.03 for HF/C, and p = 0.002 for HF/HF, Fig 5C right panel). No differences were seen in *Gas6* expression levels. Expression levels of *Ggcx* were significantly raised in the HF/HF group compared to the other three diet groups (all p<0.001, Fig 5E right panel). *Vkor* expression levels were raised in all diet groups compared to controls (p = 0.008 for HF/C, p = 0.04 for C/HF, and p<0.001 for HF/HF, Fig 5F right panel). Levels were significantly higher in HF/HF offspring compared to HF/C (p = 0.003) and C/HF (p = 0.001) offspring (Fig 5F right panel).

### Correlation of Gene Expression to Bone Structure

#### Male Offspring

The results of univariant linear regression analysis for male offspring are shown in Table 3A. *MGP* expression correlated with trabecular spacing in the femur (p = 0.02). *OCN* expression correlated with trabecular spacing in the femur (p = 0.05). *Gas6* showed negative correlation with spacing in the femur (p = 0.02), SMI (p = 0.01), and TbPF (p = 0.04). *Periostin* expression correlated with femoral trabecular spacing (p = 0.009) and also showed significant multivariant correlation for femoral bone parameters (p = 0.02) and with offspring diet (p = 0.03). *Ggcx* expression correlated with femoral trabecular spacing (p = 0.001), bone volume to total volume ratio (p = 0.007). *Vkor* expression correlated with femoral trabecular spacing (p = 0.001) and SMI (p = 0.02). Only *Gas6* showed correlation with vertebral bone parameters, with expression levels negatively correlating with trabecular separation (p = 0.01, Table 3A).

**Table 3:** Linear regression analysis between femoral vitamin K-dependent protein expression levels and bone structural factors of the femur or 3^rd^ lumbar vertebra.

A. Males																			
Site	Factor	*Mgp*			*Osteocalcin*			*Gas6*			*Periostin*			*Ggcx*			*Vkor*		
		β	r^2^	p	β	r^2^	p	β	r^2^	p	β	r^2^	p	β	r^2^	p	β	r^2^	p
Femur	Bone volume	-0.23	0.06	ns	-0.03	0.00	ns	-0.28	0.08	ns	-0.16	0.02	ns	-0.25	0.06	ns	-0.11	0.01	ns
	Length	-0.18	0.03	ns	-0.13	0.02	ns	-0.12	0.02	ns	-0.44	0.19	0.06	-0.30	0.09	ns	-0.18	0.03	ns
	BvTv	-0.34	0.12	ns	-0.29	0.08	ns	0.36	0.13	ns	-0.43	0.18	0.07	-0.56	0.31	0.007	-0.55	0.30	0.007
	BsBv	0.04	0.00	ns	0.03	0.00	ns	-0.03	0.00	ns	0.01	0.00	ns	0.17	0.03	ns	0.08	0.01	ns
	TbTh	-0.02	0.00	ns	-0.11	0.01	ns	-0.22	0.05	ns	-0.03	0.00	ns	-0.20	0.04	ns	-0.02	0.00	ns
	TbSp	0.53	0.29	0.02	0.44	0.19	0.05	-0.50	0.25	0.02	0.58	0.34	0.009	0.64	0.40	0.001	0.63	0.40	0.001
	SMI	0.29	0.09	ns	0.11	0.01	ns	-0.53	0.29	0.01	0.23	0.05	ns	0.38	0.15	0.08	0.47	0.22	0.02
	TbPF	0.20	0.04	ns	0.07	0.00	ns	-0.45	0.20	0.04	0.11	0.01	ns	0.32	0.10	ns	0.33	0.11	ns
	MidWT	-0.39	0.15	ns	-0.16	0.03	ns	-0.16	0.03	ns	-0.10	0.01	ns	-0.13	0.02	ns	-0.12	0.02	ns
	MidD	0.00	0.00	ns	0.02	0.00	ns	-0.40	0.16	0.08	0.02	0.00	ns	0.11	0.01	ns	0.16	0.02	ns
	Multivariant	F(10,4) = 3.57		ns	F(10,4) = 1.16		ns	F(10,7) = 2.32		ns	F(10,4) = 9.71		0.02	F(10,6) = 1.28		ns	F(10,7) = 2.55		ns
	+ Maternal diet	F(11,3) = 2.43		ns	F(11,3) = 1.15		ns	F(11,6) = 1.88		ns	F(11,3) = 6.74		0.07	F(11,5) = 1.08		ns	F(11,6) = 2.16		ns
	+ Offspring diet	F(11,3) = 2.52		ns	F(11,3) = 0.90		ns	F(11,6) = 2.30		ns	F(11,3) = 11.99		0.03	F(11,5) = 1.83		ns	F(11,6) = 3.39		0.07
Vertebra	Length	0.13	0.02	ns	-0.04	0.00	ns	-0.30	0.09	ns	-0.14	0.02	ns	0.02	0.00	ns	0.32	0.10	ns
	BvTv	-0.16	0.03	ns	-0.04	0.00	ns	0.39	0.16	ns	0.07	0.01	ns	-0.00	0.00	ns	0.03	0.00	ns
	BsBv	0.05	0.00	ns	-0.01	0.00	ns	-0.16	0.03	ns	-0.13	0.02	ns	-0.14	0.02	ns	-0.27	0.07	ns
	TbTh	-0.14	0.02	ns	-0.15	0.02	ns	0.01	0.00	ns	-0.08	0.01	ns	0.03	0.00	ns	0.20	0.04	ns
	TbSp	0.24	0.06	ns	0.05	0.00	ns	-0.55	0.31	0.01	-0.01	0.00	ns	0.09	0.01	ns	0.20	0.04	ns
B. Females																			
Site	Factor	*Mgp*			*Osteocalcin*			*Gas6*			*Periostin*			*Ggcx*			*Vkor*		
		β	r^2^	p	β	r^2^	p	β	r^2^	p	β	r^2^	p	β	r^2^	p	β	r^2^	p
Femur	Bone volume	-0.12	0.02	ns	0.44	0.19	0.05	-0.18	0.03	ns	-0.43	0.18	ns	0.07	0.01	ns	0.46	0.21	0.06
	Length	-0.27	0.07	ns	0.36	0.13	ns	0.01	0.00	ns	0.34	0.11	ns	0.05	0.00	ns	0.13	0.02	ns
	BvTv	0.03	0.00	ns	0.40	0.16	0.08	-0.31	0.09	ns	-0.20	0.04	ns	-0.03	0.00	ns	0.18	0.03	ns
	BsBv	-0.17	0.03	ns	-0.52	0.28	0.02	0.29	0.08	ns	0.39	0.15	ns	-0.14	0.02	ns	-0.38	0.14	ns
	TbTh	0.19	0.04	ns	0.43	0.18	0.05	-0.14	0.02	ns	-0.31	0.09	ns	0.20	0.04	ns	0.29	0.09	ns
	TbSp	0.01	0.00	ns	-0.21	0.04	ns	0.03	0.00	ns	-0.53	0.28	0.02	-0.22	0.05	ns	-0.04	0.00	ns
	SMI	0.05	0.00	ns	-0.23	0.05	ns	0.15	0.02	ns	0.21	0.04	ns	0.32	0.10	ns	-0.19	0.04	ns
	TbPF	0.00	0.00	ns	-0.37	0.14	ns	0.39	0.16	ns	0.48	0.23	0.03	0.09	0.01	ns	-0.24	0.06	ns
	MidWT	-0.45	0.20	0.06	0.05	0.00	ns	-0.49	0.24	0.03	-0.42	0.18	ns	-0.47	0.22	0.03	-0.10	0.01	ns
	MidD	0.50	0.25	0.03	0.58	0.34	0.006	0.13	0.02	ns	-0.22	0.05	ns	0.54	0.29	0.009	0.77	0.59	<0.001
	Multivariant	F(10,4) = 0.82		ns	F(10,7) = 0.85		ns	F(10,6) = 0.64		ns	F(10,4) = 1.08		ns	F(10,7) = 2.29		ns	F(10,5) = 1.57		ns
	+ Maternal diet	F(11,3) = 0.68		ns	F(11,6) = 1.71		ns	F(11,5) = 1.49		ns	F(11,3) = 1.72		ns	F(11,6) = 1.78		ns	F(11,4) = 1.42		ns
	+ Offspring diet	F(11,3) = 1.41		ns	F(11,6) = 2.18		ns	F(11,5) = 0.83		ns	F(11,3) = 0.74		ns	F(11,6) = 2.59		ns	F(11,4) = 1.15		ns
Vertebra	Length	0.04	0.00	ns	0.33	0.11	ns	0.28	0.08	ns	-0.22	0.05	ns	0.27	0.08	ns	0.35	0.12	ns
	BvTv	0.31	0.10	ns	0.27	0.07	ns	-0.20	0.04	ns	-0.59	0.35	0.02	0.09	0.01	ns	0.32	0.11	ns
	BsBv	-0.38	0.15	ns	-0.52	0.27	0.02	0.09	0.01	ns	0.71	0.51	0.002	-0.15	0.02	ns	-0.46	0.21	0.07
	TbTh	0.34	0.11	ns	0.50	0.25	0.03	-0.08	0.01	ns	-0.77	0.59	<0.001	0.05	0.00	ns	0.44	0.19	0.08
	TbSp	-0.23	0.06	ns	-0.06	0.00	ns	0.30	0.09	ns	0.32	0.10	ns	-0.19	0.04	ns	-0.36	0.13	ns

Abbreviations are BvTv: trabecular bone volume to total volume, BsBv: Bone surface to bone volume ratio, TbTh: Trabecular thickness, TbSp: Trabecular spacing, SMI: Structural model index, TbPF: Trabecular pattern factor, MidWT: Midshaft wall thickness, MidD: Midshaft diameter. For Diet, β value is based on high fat diet having a higher value than control diet.

#### Female Offspring

The results of univariant linear regression analysis for female offspring are shown in Table 3B. *MGP* expression correlated with femoral midshaft diameter (p = 0.03). *OCN* expression correlated with femoral bone volume (p = 0.05), femoral trabecular thickness (p = 0.05), bone surface to volume ratio (p = 0.02), femoral midshaft diameter (p = 0.006). *Gas6* expression negatively correlated with femoral midshaft wall thickness (p = 0.03). *Periostin* expression negatively correlated with femoral trabecular spacing (p = 0.02) and TbPF (p = 0.03). *Ggcx* expression negatively correlated with femoral midshaft wall thickness (p = 0.03) and correlated with femoral midshaft diameter (p = 0.009). *Vkor* expression correlated with femoral midshaft diameter (p<0.001). Vertebral bone parameters showed significant results with *OCN*, with a negative correlation compared to bone surface to volume ratio (p = 0.02), and positive correlation with trabecular thickness (p = 0.03, Table 3B). *Periostin* expression correlated with vertebral bone surface to volume ratio (p = 0.002), negatively correlated with bone volume to total volume ratio (p = 0.02) and vertebral trabecular thickness (p<0.001, Table 3B).

## Discussion

To our knowledge, this is the first study to determine the long-term effects of the maternal diet on the expression levels of VKDPs from the offspring in older age. This study extends the analysis of VKDPs previously made in 6 week old offspring [27], when the mice have just become sexually mature, into offspring of 30 weeks of age, to assess the longer term effects of a HF diet on VKDP expression. Previously, no correlations were found between *OCN*, *Periostin*, *Gas6*, or *Vkor* and any of the body or bone parameters (listed in Tables 1 and 3) in 6 weeks old offspring [27]. Therefore, the correlations found with *OCN*, *Periostin* and *Vkor*, in the 30 week animals studied here, are a consequence of age. In contrast, the correlations found with *MGP* in 6 week old offspring [27] have largely disappeared with age.

The *MGP* expression level in the aorta was reduced in all diet groups at 30 weeks of age compared to 6 weeks of age (S1 Fig). Furthermore, the *MGP* expression level in the femur was reduced in all diet groups at 30 weeks of age compared to 6 weeks of age (except male HF/HF, where the level was maintained) (S2 Fig). The lower expression levels may be due to a reduction in growth plate thickness, calcification of the bone, or other parameters related to maturation of bone, and hence a reduction in the requirement to prevent calcification of chondrocytes in growing bone.

While 6 week old offspring showed no correlations with *OCN* expression and any of the parameters listed in Tables 1 and 3, probably due to an absence of any differences in fat volumes at this timepoint [27], the expression levels of *OCN* in the femur were on average 50 fold higher at 6 weeks of age than at 30 weeks of age (S3 Fig). Interestingly, at 30 weeks of age *OCN* expression correlated with body composition, specifically fat volume (and additionally tissue volume in males), but surprisingly not bone volume, as would be expected with OCN being produced by osteoblasts.

Work from the Karsenty group recently showed that insulin resistance in bone, due to increased levels of free saturated fatty acids, contributed to the deleterious consequences of a long-term HF diet on whole-body glucose homeostasis, in part as a consequence of decreased osteocalcin activity [28]. This would appear to be the case in the C/HF male group (Karsenty’s group fed male mice a HF diet from 6 to 18 weeks of age). However, female offspring C/HF and HF/HF groups and male HF/HF offspring appear to show the opposite effect, with increasing *OCN* levels. Hence, OCN may play a different role in females, and *in utero* exposure to a HF diet in males may alter their response to a postnatal HF diet.

No correlations were found between bone parameters and *periostin* expression in 6 week old samples [27]. Hence, the correlations reported here have appeared with age. However, these correlations are sex specific. For example, *periostin* expression only correlated to body composition in male offspring and not female offspring. Furthermore, there was a positive correlation with trabecular spacing in males yet a negative correlation with trabecular spacing in female offspring. Periostin expression also appeared to be site specific as bone (femoral) expression correlated with a weak bone phenotype in the vertebra, but not the femur, and only in female offspring. There is increasing evidence that periostin plays a role in determining bone adaptations during loading and unloading such that increasing *periostin* expression increases bone strength; at least in male mice [29,30]. Certainly, in the males studied here, the heavier HF/HF offspring had higher femoral expression levels of *periostin* and increased cortical bone than lighter controls. *Periostin* expression also correlated to body mass and composition. However, this did not occur with our female samples, where there was also no correlation between body mass and composition and *periostin* expression. In addition, male HF/C group had similar mass and femoral bone characteristics to male controls, yet had significantly lower *periostin* expression. Furthermore, the trabecular structure in HF/HF offspring would be expected to be weaker than controls due to high trabecular spacing with constant trabecular thickness. These data suggest diet and sex have altered the response to, or outcome of, *periostin* expression potentially due to altered vitamin K availability with increased fat mass.

All groups showed lower level of *Ggcx* expression in the femur at 30 weeks compared to 6 weeks of age (average 5 times lower) (S4 Fig). These lower expression levels would indicate a lower level of vitamin K carboxylation within the femur in 30 week offspring compared to 6 week old offspring. *Ggcx* and *Vkor* expression levels were correlated to fat volume. This suggests that with increasing obesity, the availability of vitamin K is reduced and hence gene expression levels are increased in an attempt to produce sufficient active VDKP and active vitamin K. Without excess vitamin K, over expression of *Vkor* increases the proportion of carboxylated to undercarboxylated VKDP as the rate of vitamin K hydroquinone production rather than the rate of vitamin K–dependent carboxylation can be the rate-limiting step for *in vivo* vitamin K–dependent protein carboxylation. Co-over expression with *Ggcx* can increase this proportion further [31]. Alternatively, Shiba et al. suggest Ggcx in osteoblasts regulates glucose metabolism in mice [32]. The serum undercarboxylated OCN and carboxylated OCN levels were observed to be substantially increased and decreased, respectively, in Ggcx knockout mice in comparison to controls. The authors suggested that Ggcx expressed in osteoblasts was critical for the maintenance of blood glucose and white adipose tissue, recently confirmed by Ferron et al.[33]. These findings are in agreement with our data and the correlation between *Ggcx* expression levels and fat volume. In addition, as Shiba et al. [32] showed Ggcx may alter serum OCN levels, this could account for the correlation between *OCN* and fat volume we have observed. Unfortunately, it was not possible to measure serum levels of VKDP’s in this study.

Male offspring showed differences in femoral bone structure particularly in the HF/HF diet group compared to controls. In addition, the bone density data suggested the HF/HF group had overall less trabecular bone and more cortical bone (area under the curve for each appropriate density range). These results can be accounted for by the increased trabecular spacing in the femur, and increased femoral diameter. Although the latter measurement was not statistically significant between the groups, these analyses were performed on a small section of the femur, whereas the density plots represent the entire femur. While few differences were seen in trabecular structural parameters in female offspring diet group, the maternal HF groups showed increased femoral bone volume, increased midshaft diameter that also correlated with increased mass. Thus, although the femur from the heavier offspring may be able to support the mass of the animal due to increased cortical thickness, the femur would be no stronger with regard to trabecular strength than lighter animals and therefore could be more susceptible to fracture.

Similarly, most of the differences in vertebral bone structure were seen in female offspring. Trabecular thickness within the vertebral body increased with mass, except in the HF/HF group, which despite having greater mass than the C/HF group, showed a trabecular thickness significantly less than the C/HF group. Given the reduced trabecular thickness and increased mass of the HF/HF group, these animals would be expected to be more susceptible to vertebral fracture than the C/HF group. For the whole vertebra, female offspring from HF-fed dams showed a higher proportion of lower density trabecular bone. Male offspring showed few differences, although offspring from HF-fed dams showed significantly increased trabecular spacing within the vertebral body. Consequently, the femur and vertebra in female offspring appear to be more affected by the maternal HF diet than these bones in male offspring, as is seen with a maternal low protein diet [34].

In support of the HF/HF group being potentially more at risk of bone fracture than the C/HF group, the majority of the VKDPs, *Ggcx* and *Vkor* showed differences in expression in the femur between these two offspring groups, with the HF/HF group displaying higher expression levels, although, interestingly, this occurred in males more often than females. Few differences, either within bone structure parameters or gene expression, were seen between the C/C and HF/C groups, suggesting the postnatal HF diet induced a greater impact in comparison to maternal diet affects, as seen in bone marrow adiposity [35].

It is possible that alterations in VKDP, Ggcx and Vkor gene expression are due to epigenetic mechanisms caused by the high fat diet at times when the offspring genome is highly plastic, such as *in utero* and early postnatal life. Indeed, a high fat diet can alter all aspects of epigenetic modification so far studied, including DNA methylation, histone modification, and microRNAs, in a variety of tissues, and in both humans and animal models [36]. For the data presented here, the response was also tissue specific, as femoral MGP expression was positively correlated to fat mass in males, yet aortic MGP expression was negatively correleted (data not shown) in the same animals. Hence, further study is required in order to determine if the same epigenetic control is utilised in different tissues, for example, is microRNA control more prevalent in aortic tissue than femoral tissue. Gene expression of Ggcx and Vkor showed interesting results. There were also differences in gene expression between the two “switched” diets (C/HF and HF/C) demonstrating the effect of postnatal plasticity, although the differences in gene expression between the C/HF and HF/HF groups suggested maintenance of the high fat diet from *in utero* through the postnantal period had greater influence on gene expression, possibly by making any epigenetic modification more stable though later life [37].

In summary the current studies demonstrate the importance of maternal nutrition during pregnancy and the effects on skeletal bone development of the offspring. Furthermore, the modulation of vitamin k dependent proteins observed in both male and female offspring indicates the need to further elucidate the mechanisms by which the programmed skeletal changes observed occur with implications for our understanding of bone physiology and ageing and risk to chronic diseases in later life.

## Supporting Information

S1 Fig MGP expression level in Aorta from 6 and 30 week old offspring. (DOCX)

S2 Fig MGP expression level in Femur from 6 and 30 week old offspring. (DOCX)

S3 Fig Osteocalcin expression level in Femur from 6 and 30 week old offspring. (DOCX)

S4 Fig Ggcx expression level in Femur from 6 and 30 week old offspring. (DOCX)
