# Supplementary material for: Maternal High Fat Diet Affects Offspring’s Vitamin K-Dependent Proteins Expression Levels
Source: PLoS One. 2015 Sep 18;10(9):e0138730. doi: 10.1371/journal.pone.0138730 (PMC4575216; doi:10.1371/journal.pone.0138730)

At the start of pregnancy dams were fed either a control (C) diet or a high-fat (HF) diet. These C or HF diets were continued throughout pregnancy and lactation until the offspring had been weaned. The weaned offspring were then further subdivided to provide each parental dietary group with offspring that had been fed either the control (C/C or HF/C groups) or the high-fat diet (C/HF or HF/HF groups). Gene expression was determined by qPCR.

**Figure S1. MGP expression level in Aorta from 6 and 30 week old offspring.**


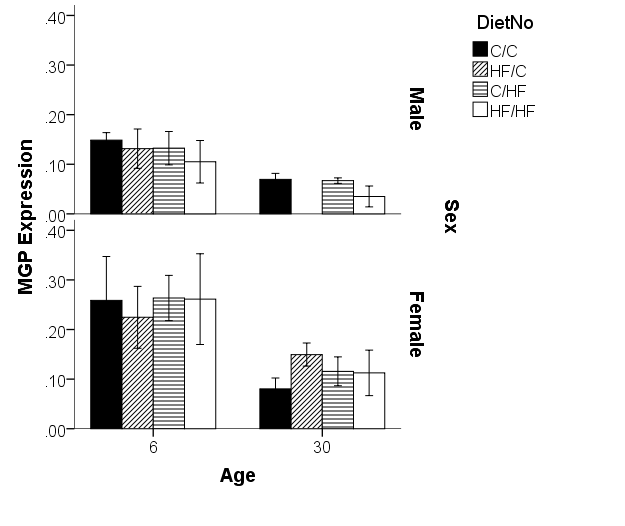

Supplement: S1 Fig — (DOCX) [file pone.0138730.s001.docx]
